# Bacterial Responses and Genome Instability Induced by Subinhibitory Concentrations of Antibiotics

**DOI:** 10.3390/antibiotics2010100

**Published:** 2013-03-14

**Authors:** Luisa Laureti, Ivan Matic, Arnaud Gutierrez

**Affiliations:** Faculté de Médecine Paris Descartes, 24 rue du Faubourg Saint-Jacques, INSERM U1001, Université Paris Descartes, Sorbonne Paris Cité, 75014 Paris, France; E-Mails: luisa.laureti@inserm.fr (L.L.); gutierrez.arnaud@gmail.com (A.G.)

**Keywords:** antibiotics, resistance, stress response, mutagenesis

## Abstract

Nowadays, the emergence and spread of antibiotic resistance have become an utmost medical and economical problem. It has also become evident that subinhibitory concentrations of antibiotics, which pollute all kind of terrestrial and aquatic environments, have a non-negligible effect on the evolution of antibiotic resistance in bacterial populations. Subinhibitory concentrations of antibiotics have a strong effect on mutation rates, horizontal gene transfer and biofilm formation, which may all contribute to the emergence and spread of antibiotic resistance. Therefore, the molecular mechanisms and the evolutionary pressures shaping the bacterial responses to subinhibitory concentrations of antibiotics merit to be extensively studied. Such knowledge is valuable for the development of strategies to increase the efficacy of antibiotic treatments and to extend the lifetime of antibiotics used in therapy by slowing down the emergence of antibiotic resistance.

## 1. Introduction

With the early breakthroughs of Fleming and Waksman, the discovery of novel natural antibacterial compounds with important pharmaceutical applications revolutionized medicine and experienced an exponential phase, especially between 1950s and 1960s, the so called “Golden Age”. At that time, the potential problem of antibiotic resistance was unfortunately underestimated, in spite of the fact that the first multi-drug resistant strain appeared already in 1955 (reviewed by [1]). It soon became evident that bacterial resistance can be acquired through mutations or horizontal gene transfers [1]. Today, the worldwide spread of antibiotic resistance is a major healthcare and economic problem because it directly challenges our ability to treat infectious diseases. To extend the lifetime of current and future antibiotic-based therapies, it is increasingly urgent to enlarge our knowledge of how new antibiotic resistances emerge and spread in bacterial population. In this review, we will firstly focus on the bacterial responses to subinhibitory concentrations of antibiotics, with particular emphasis on the induction of the SOS and RpoS regulons. We will then describe how subinhibitory concentrations of antibiotics promote genetic variation by increasing the rates of horizontal gene transfer and mutations. Finally, we will discuss how these molecular mechanisms are directly responsible for the emergence and the spread of resistance determinants.

## 2. Antibiotics: Killing or Signaling Molecules?

Antibiotics are low molecular weight molecules (<3,000 Da) found in all kinds of terrestrial and marine environments. They are mainly produced by fungi (60%) and actinomycetes (30%), especially by the genus *Streptomyces*, but also by other bacteria (10%), such as *Bacillus*, *Pseudomonas*, Myxobacteria and Cyanobacteria [2]. Antibiotics are metabolites often produced by organisms that undergo morphological differentiation or by organisms that experience nutrient limiting conditions. This production is usually triggered by specific cellular signaling. Antibiotics can be grouped on the basis of their chemical structures, which are extremely varied and complex, or of their mechanism of action [3]. The targets of antibiotics might be either cellular structures or enzymes. The most common mechanisms of action involve the inhibition of the bacterial cell wall biosynthesis (e.g., β-lactam, glycopeptides); the inhibition of protein, RNA or DNA synthesis (macrolides, ansamycins, quinolones, respectively); and the damage of cell membranes (polymyxins). Antibiotics can also be divided in two major classes according to their biological effects: bactericidal compounds that kill bacteria (e.g., β-lactam, fluoroquinolones and aminoglycosides) and bacteriostatic compounds that inhibit bacterial growth (e.g., macrolides, tetracyclines). Recently, a common mechanism of cell death has been described, which is distinct from the classical described targets and shared by several classes of bactericidal antibiotics [4]. This mechanism involves the stimulation of endogenous reactive oxygen species (ROS) production, which damages lipids, proteins and DNA, thus leading to a sort of programmed cellular death that shares several characteristics with apoptosis [5,6]. 

Two hypotheses have been formulated on the possible natural role of antibiotics. The first postulates that antibiotics are biological weapons that protect the producer strain from bacterial competitors present in the same environment, in particular under stress conditions such as nutrient starvation [7]. For example, filamentous fungi and actinomycetes have a non-motile and saprophytic life cycle in complex habitats, such as the terrestrial soil, where competition with other inhabitants is high. Nevertheless, few examples in literature have demonstrated the role of these microbial products as antibacterials in the natural environments [8,9].

The second hypothesis that suggests that antibiotics are involved in signaling mechanisms is supported by several lines of evidence. Many studies have demonstrated that exposure to subinhibitory concentrations of antibiotics induces changes in the expression profile of a wide range of genes in many different bacterial species, resulting in different phenotypes [10,11]. In addition, *in situ*, antibiotics are produced by bacteria and fungi at a very low concentration that would not have any lethal effects. 

Subinhibitory concentrations of antibiotics have also been found in aquatic and terrestrial environment due to their use in human and veterinary medicine and in agriculture [12]. Because antibiotics are not always well metabolized in human and animal bodies, they are excreted in active form. A similar fate is shared by the antibiotics used in agriculture. In the soil or in sewage, antibiotics are not fully biodegraded or removed by chemical treatments. For all these reasons, active antibiotics end up polluting soil, surface and ground water. Hence, we live surrounded by subinhibitory concentrations of antibiotics that can have important consequences on the resident microbial communities. 

The initial definition of antibiotic given by Waksman, *i.e*., “a natural chemical substance, derived from living microorganisms, which has the capability to inhibit growth or even destroy other microorganisms without harming the eukaryotic host” does not encompass chemically synthesized antibiotics, such as quinolones, sulphonamides or oxazolidinones. In this review we will indiscriminately consider the effects of natural and synthetic antibiotics. 

## 3. Bacterial Responses to Subinhibitory Concentrations of Antibiotics

As discussed above, antibiotics are found in natural environments at subinhibitory concentrations. Transcriptome analyses, gene reporter fusions and classical genetic studies have shown that subinhibitory concentrations of antibiotics trigger wide changes in the transcriptional profiles and in the phenotypes of various bacterial species, such as *Escherichia coli*, *Salmonella typhimurium*, *Pseudomonas aeruginosa* or *Staphylococcus aureus *[10]. Most of the genes differentially expressed in the presence of subinhibitory concentrations of antibiotics encode functions not obviously linked with those targeted by the antibiotics. These observations support the idea that antibiotics may act as signaling molecules in nature.

Because antibiotics target essential cellular functions, it is not unexpected that even at subinhibitory concentrations they can induce different stress responses, such as the SOS response or the RpoS regulon [13,14,15]. The SOS response is induced by the presence of unprotected single-strand DNA resulting from DNA damage and DNA replication arrest. The induction of the SOS response is mediated by the autocleavage of the LexA repressor, which can be stimulated by the co-protease activity of the RecA protein when it is associated with single-strand DNA. The inactivation of LexA results in the expression of about 40 genes encoding functions mostly involved in the DNA repair [16,17]. Antibiotics such as quinolones impair the function of enzymes interacting with DNA and thus promote the formation of DNA damage and replication arrest [18]. Therefore, the SOS induction observed after quinolone treatment results directly from their mechanism of action. In *E. coli*, lethal doses of β-lactams promote the induction of the SOS response, which is mediated by the two components system DpiAB [19]. This induction is therefore independent of DNA damage unlike the SOS induction by quinolone antibiotics. Other antibiotics that do not target functions directly linked with DNA, like aminoglycosides, may induce the SOS response through the stimulation of cellular ROS production, as suggested by the work of Kohanski *et al*. [4,20]. 

We recently showed that in *E. coli*, subinhibitory concentrations of different bactericidal antibiotics stimulate the induction of the general stress response, which is controlled by the RpoS sigma factor [15]. The induction of the RpoS regulon is controlled by many different factors acting at the level of the *rpoS* gene expression, *rpoS* mRNA stability or RpoS protein translation and stability [21]. The RpoS regulon was historically linked with stationary-phase gene expression. However, today we know that this regulon may be induced by a wide range of stress conditions like heat shock, starvation, low pH or osmotic shock [21]. The alternative sigma factor RpoS is conserved in many bacterial species and controls the expression of many genes involved in cell shape determination, stress response, biofilm formation, DNA repair, metabolism or genes coding for virulence factors [22]. Therefore, the functions of the RpoS-regulated genes may have an important impact on the emergence of antibiotic resistance and on the virulence potential of stressed bacterial populations. For instance, we showed that the induction of the RpoS regulon is required for increased mutagenesis in cells treated with subinhibitory concentrations of β-lactam antibiotics [15]. This is of high relevance because even slight modifications in the mutation rates can significantly influence the evolution of antibiotic resistance [23]. 

Numerous studies on the effect of subinhibitory concentrations of antibiotics report modulation in the expression of genes coding for virulence factors, such as exo and endotoxins or adhesins, in several human pathogen species [24,25,26,27,28]. In *S. aureus *treated with different β-lactam antibiotics, promoter-*lux* reporter constructions allowed observing and quantifying the induction of the *spa*, *lukE* and *agr* genes, known to code for virulence functions [28]. Induction of virulence genes by subinhibitory concentrations of antibiotics is of high importance from a clinical point of view because it may increase virulence of pathogens and hence contribute to increased morbidity and mortality. However, subinhibitory concentrations of antibiotics can also inhibit virulence gene expression, as demonstrated by Grimwood *et al*. [25], in the case of the exotoxins production of *P. aeruginosa*. 

The majority of the studies on gene expression or on phenotypic changes induced by subinhibitory concentrations of antibiotics have been conducted in homogenous liquid environments. To extend knowledge on the effects of subinhibitory concentrations of antibiotics, it is important to perform studies in structured environments, such as biofilms, because bacteria are predominantly found in such environments in nature. For instance, Zhang *et al*. [29] have already shown that the emergence of antibiotic resistance is accelerated in structured environments. The microfluidic device used in this study consists of an interconnected multi chamber chip that favors the formation of an antibiotic gradient. This structure mimics the environmental conditions, such as the human body, where cells encounter nutrient and chemical gradients. In such a structured environment, antibiotic resistant mutants appearing in areas of low antibiotic concentration can move and overtake sensitive bacteria in areas of higher antibiotic concentration. 

In addition, among the different phenotypes induced by subinhibitory concentrations of antibiotics, the stimulation of biofilm production was observed in numerous human pathogens, such as *Staphylococcus* or *Pseudomonas* species [11]. Biofilms can directly challenge the treatment of infectious diseases by greatly reducing the antibacterial efficacy of antibiotics [30,31]. Biofilm structures are heterogeneous environments in which bacteria face gradients of physical and chemical parameters, such as nutrients, oxygen, pH. Consequently, bacteria are in distinct physiological states, which endow them with variable capacity to tolerate antibiotics. The physical barrier created by the biofilm structure can also slow the diffusion of antibiotics [32,33] and therefore can promote the appearance of zones of subinhibitory concentrations of antibiotics. Because subinhibitory concentrations of antibiotics can induce stress responses, which in turn increase the capacity of bacteria to resist to higher doses of antibiotics, a vicious circle could be created. 

The formation of biofilms is induced by different environmental signals through molecular pathways often involved in quorum sensing or second-messenger signaling [34]. Biofilm formation, induced by subinhibitory concentrations of antibiotics targeting ribosomes, such as aminoglycosides, phenicols or tetracyclines, was shown to involve cyclic-di-GMP signaling both in *P. aeruginosa *and *E. coli *[35]. In *P. aeruginosa*, biofilm induction requires the presence of an inner membrane protein, coded by the *arr* gene, containing an EAL domain [35]. The EAL domain is commonly present in enzymes involved in the degradation of the cyclic-di-GMP, thus aminoglycosides may modulate the level of this second messenger by acting on the inner membrane protein. In *E. coli*, translational inhibitors promote the induction of poly-GlcNAc through the up-regulation of the activity of the *pga* genes products [36]. As for *P. aeruginosa*, cyclic-di-GMP is essential in this process and requires the di-guanilate-cyclase activity of the enzyme YdeH. 

The stimulation of biofilm production by subinhibitory concentrations of β-lactam antibiotics has been also demonstrated in *E. coli*, *P. aeruginosa*, *S. aureus *and *Streptococcus pneumoniae *[37,38,39,40]. For example, in *E. coli*, β-lactam treatments can increase the expression of *cps *genes, which code for colonic acid production pathway, while in *P. aeruginosa*, subinhibitory concentrations of imipenem increase the production of alginate, thus favoring the formation of thicker and more robust biofilms.

Bacterial responses to subinhibitory concentrations of antibiotics raise the question of whether these responses result from a specific signal triggered by the antibiotics or whether they are only the consequences of a perturbation in the cellular homeostasis resulting from the antibiotic action. Even though the above described responses to antibiotics, like SOS, RpoS regulon or biofilm formation, are induced by a variety of different stresses, the presence of signaling pathways dedicated to respond specifically to antibiotics cannot be excluded. 

## 4. Subinhibitory Concentrations of Antibiotics Enhance Horizontal Gene Transfer

Subinhibitory concentrations of antibiotics strongly stimulate the transfer of mobile elements such as transposons, insertion sequences (ISs), integrons, integrating conjugative elements (ICEs) or pathogenicity islands (PIs), through transformation, conjugation or transduction [41]. These mobile elements can contain genes coding for different antibiotic resistance, heavy metal resistance or virulence factors, thus conferring a multi-resistant phenotype to the host cell. For example, it was shown that subinhibitory concentrations of β-lactam antibiotics enhanced the rate of conjugative plasmid transfer in *S. aureus *[42] and that pre-treatment of donor *Bacteroides* cells with tetracycline enhanced the conjugal transfer of different ICEs [43,44].

The soil microcosm is one of the largest and diverse reservoirs for antibiotic resistant determinants in the form of mobile elements [41,45,46]. Subinhibitory concentrations of antibiotics found in the terrestrial environment through manure fertilization or sewage exposure may significantly contribute to the mobilization of these elements. The presence of antibiotics in the soil increases the horizontal gene transfer and generates diversity in the mobile elements [47]. In a soil treated with sulfadiazine, Heuer and colleagues found a novel low G+C content plasmid harbouring different antibiotic resistance genes, including *tet*(X) able to confer resistance to the third-generation of tetracyclines. The putative hosts for this new plasmid are *Actinobacter* spp, of which *A. baumannii* is one of the recent emerging multi-drug resistance strains in hospitals. 

The SOS response can promote the expression of genes involved in horizontal gene transfer. Subinhibitory concentrations of some antibiotics induce the SOS response, thus indirectly inducing horizontal transfer. The frequency of transfer of the SXT ICE of *Vibrio cholerae *is increased more than 300-fold when the *E. coli *donor cell is grown in the presence of subinhibitory concentrations of mitomycin C or ciprofloxacin, which both induce the SOS response and consequently genes necessary for the SXT transfer [48]. Similarly, subinhibitory concentrations of fluoroquinolones induce the SOS response in *S. aureus* and promote the replication and transfer of the pathogenicity island SaPIbov1, as well as the induction of the prophage encoding Shiga toxin [49]. In addition, the SOS response, induced by subinhibitory concentrations of antibiotics, was recently demonstrated to promote the expression and recombination of integrons [50]. However, the SOS induction by subinhibitory concentrations of antibiotics is not the only known mechanism that stimulates horizontal gene transfer. For example, subinhibitory concentrations of different antibiotics induce genetic transformation in the naturally competent *S. pneumoniae*, which lacks a SOS-like response [51].

In the last decade, the intestine, an enormously dense and diverse microcosm, has come to the fore as another important reservoir of antibiotic resistance determinants [52]. The current metagenomic studies of the mammalian gut microbiome highlight the abundance and diversity of mobile elements carrying resistance determinants. In addition, previous works have demonstrated that intra and inter-species horizontal gene transfer occurs between commensal and pathogenic bacteria resident in or passing through the gut. In the human and animal intestine, bacteria can encounter a gradient of antibiotic concentrations because of therapeutic use/abuse with direct and important consequences [52]. Since the 90’s, several studies have demonstrated that subinhibitory concentrations of antibiotics induce the transfer of conjugative plasmids harboring antibiotic resistance in the digestive tracts of gnobiotic mice [53,54]. However, no evidence yet demonstrates that horizontal gene transfer is enhanced by the presence of low antibiotic concentrations in the human intestine, but this is likely in our view. 

## 5. Increasing Mutation Rates in Response to Subinhibitory Concentrations of Antibiotics

**Figure 1:**
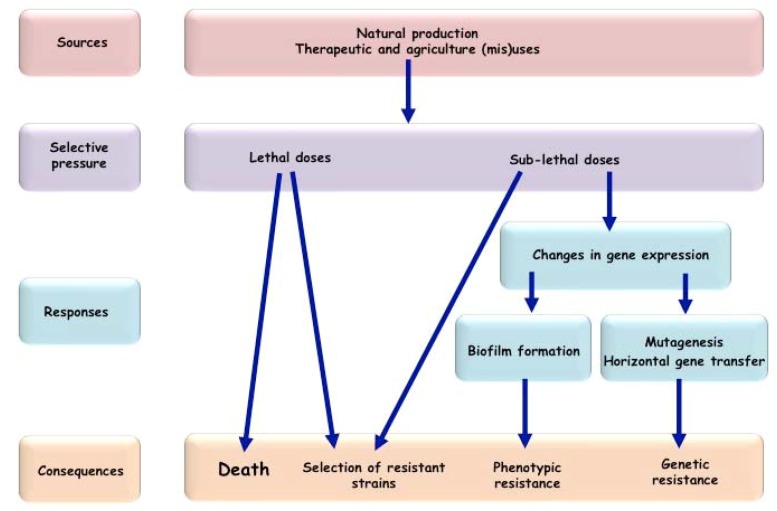
Impact of antibiotics on bacterial population.

Over the years it has become evident that antibiotics can directly affect the rate of emergence of antibacterial resistance determinants in a bacterial population [55,56,57]. Lethal doses of antibiotics select for pre-existing resistant strains. Sub-lethal doses can also select for pre-existing resistant strains [58], but in addition, they can favor the emergence of new resistant determinants by increasing the mutation rate and their spread through the stimulation of the horizontal gene transfer [59] (Figure 1). Emergence of resistance through mutations is especially relevant for resistance resulting from the modifications of the antibiotic targets, such as quinolone or rifampicin families, but also for the evolution of genes conferring resistance through enzymatic antibiotic modifications, such as β-lactam and cephalosporins. Molecular mechanisms by which subinhibitory concentrations of several unrelated classes of antibiotics induce mutagenesis have been characterized in different bacterial species, but most exhaustively in *E. coli* (Table 1).

The most studied mutagenic antibiotics belong to the quinolone family, such as ciprofloxacin or nalidixic acid. The primary targets of quinolones are type II DNA topoisomerase, the DNA gyrase and topoisomerase IV coded by the *gyrA*, *gyrB* and *parC*, *parE* genes, respectively [18]. The inhibition of the “ligase activity” of these enzymes leads to the formation of double-strand breaks in DNA, potentially resulting in mutations and cell death [60]. In *E. coli*, the repair of double-strand breaks requires the homologous recombination pathways: RecBCD or RecFOR [61]. Processing double-strand breaks is also known to trigger the induction of the SOS response [62]. The SOS stress response controls the expression of the error-prone DNA polymerases PolII, PolIV and PolV coded respectively by the *polB*, *dinB* and *umuCD* genes [16]. Cirz and coworkers [63] found that subinhibitory concentrations of ciprofloxacin promote mutagenesis through the same pathways as UV, X or gamma rays, *i.e*., through induction of the error-prone DNA polymerases. These polymerases are required for the emergence of ciprofloxacin-induced resistant mutants *in vitro* and *in vivo *mouse model. As quinolones promote DNA damage, it is not surprising that they can promote mutagenesis by the induction of genes coding for the error-prone DNA polymerases, thus provoking the emergence of antibiotic resistant mutations. This also applies to folate inhibitor antibiotics, like sulfonamides, which by perturbing the nucleotide pool increase the error rate of the DNA polymerases and consequently increase the rate of emergence of antibiotic resistance [64]. 

**Table 1:** Molecular mechanisms involved in antibiotic-induced mutagenesis in different bacterial species.

Organisms	β-lactam	Quinolones	Aminoglycosides	Tetracyclines
*Escherichia coli*	ROS species [55] *recA*, *dinB* [14,65] *rpoS* [15]	ROS species [55] *recA*, *recBCD*,*lexA* [14,63] *polB*, *dinB*, *umuCD * [66]	ROS species [55] *recA*, *recBCD*, *ruvABC* [67]	recA [14]
*Streptococcus pneumoniae*			*dinB*-independent [68]	
*Streptococcus uberis*		*umuC*-independent [69]		
*Vibrio* * cholerae*	*lexA *[13]	*lexA *[13]	*lexA *[13]	*lexA *[13]
	*rpoS, dinB *[15]			
*Staphylococcus aureus*	ROS species [55]			
*Pseudomonas aeruginosa*	*rpoS, dinB *[15]			

Mutagenesis is not only induced by antibiotics that target functions related to DNA replication or DNA metabolism, but also by antibiotics targeting ribosomes (*i.e*., aminoglycosides or tetracyclines) or cell wall synthesis (*i.e*., β-lactam antibiotics) [13,14,15,55]. Ribosome-targeting antibiotics promote mutagenesis in different bacterial species [13,55]. However, the molecular factors involved have been investigated in detail only for streptomycin. Streptomycin is a bacteriostatic antibiotic that targets the 16S rRNA of the 30S subunit of the ribosome, promoting mistranslation. In *E. coli*, mistranslation induced by streptomycin is a key factor for the increase in mutagenesis [70]. One possible explanation is that mistranslation generates aberrant proteins, among which a mutator allele of the proofreading subunit of the replicative polymerase, hence increasing the error rate during DNA replication. This process is closely related to mistranslation-induced mutagenesis caused by the error-prone alleles coding for tRNA or 16S rRNA [67,71,72,73]. Subinhibitory concentrations of other aminoglycosides, like gentamicin or kanamycin, also increase mutagenesis in *E. coli* and in other species [15,55]. The molecular mechanisms involved in this process can be mistranslation as for streptomycin. However, their ability to increase ROS production could also be responsible for the increase in mutagenesis, as we will discuss below. 

β-lactam antibiotics exhibit a mutagenic activity in different unrelated bacterial species such as *E. coli*, *V. cholerae* and *P. aeruginosa* [15]. In all three species, mutagenesis induced by subinhibitory concentrations of β-lactam antibiotics almost exclusively depends on the PolIV activity [15,65]. Although an increase in the PolIV cellular amount was observed in cells treated with subinhibitory concentrations of ampicillin, the expression of the *dinB* gene was not induced and mutagenesis did not depend upon the SOS repressor LexA cleavage [15]. *In vitro* and *in vivo* studies showed that PolIV has a mutagenic activity by generating base substitutions and frameshifts or by incorporating oxidized nucleotides [74,75,76,77]. Our analysis of the mutational spectrum of cells treated with a subinhibitory concentration of ampicillin showed indeed that the PolIV promotes a wide variety of mutations including IS mobility [15]. 

**Figure 2:**
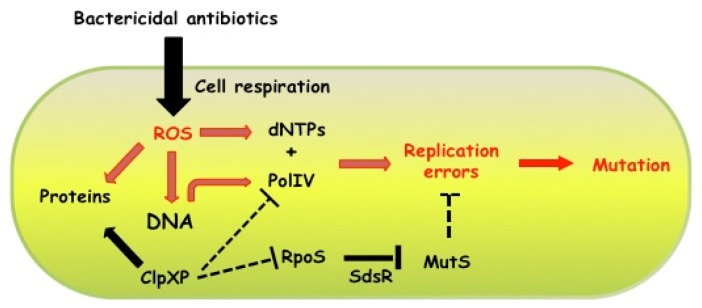
Schematic representation of how *Escherichia coli* cells modulate mutation rates in response to subinhibitory concentrations of bactericidal antibiotics. Bactericidal antibiotics, like ampicillin, induce ROS production by stimulating cellular respiratory activity. ROS damage all cellular macromolecules, thus promoting, for example, protein oxidation, DNA replication arrest and oxidation of dNTPs pool. In the presence of subinhibitory concentration of ampicillin, the amount of RpoS and PolIV proteins is increased, most likely because the ClpPX protease-chaperon complex, which degrades both RpoS and PolIV, becomes titrated by an increased amount of oxidized proteins. At the same time, the arrest of the DNA replication forks together with higher level of the PolIV error-prone DNA polymerase favors the incorporation of oxidized dNTPs into the DNA, which eventually results in generation of mutations. However, antibiotic-increased mutagenesis is possible only because the mismatch repair system is not able to repair all the PolIV-generated mutations in ampicillin treated cells. The reduction of mismatch repair activity in antibiotic-treated cells is mediated by SdsR, an RpoS-controlled small RNA, which interacts with the *mutS* mRNA [15].

We have recently demonstrated that the induction of the RpoS regulon is also a key factor in the increase of mutagenesis induced by subinhibitory concentrations of ampicillin. Among the RpoS-controlled genes induced by ampicillin, *sdsR*, which encodes a small RNA [78], has been proposed to negatively control the level of the MutS protein [15]. Hence, our study provides the first example of a molecular mechanism that directly controls the replication fidelity, thus the mutation rate, in response to an antibiotic-induced stress (Figure 2). The molecular factors required for the mutagenesis induced by β-lactams, *i.e*., RpoS, DNA PolIV induction and MutS depletion, were shown to be conserved among *E. coli*, *V. cholerae* and *P. aeruginosa *[15]. 

Kohanski and co-workers [4] showed that different bactericidal antibiotics promote ROS formation at both lethal and subinhibitory concentrations [4]. In *E. coli*, the amount of ROS induced in the presence of subinhibitory concentrations of bactericidal antibiotics is correlated with the mutagenic effect of the antibiotics [55]. ROS are known to damage intracellular macromolecules, as lipids, proteins and DNA. However, we showed that the increase in ROS production is required but not sufficient for the increase in mutagenesis. In an *rpoS* deficient mutant treated with a subinhibitory concentration of ampicillin, the increase in ROS levels is similar as in the wild-type strain, without an accompanying increase in mutagenesis although both strains possess a functional *dinB* gene [15]. As described above, an RpoS-dependent genetic regulation appears to be the key factor in antibiotic induced mutagenesis in *E. coli *(Figure 2). 

## 6. Concluding Remarks

Due to the extensive use and abuse, low concentrations of antibiotics are commonly polluting natural environments. It is becoming increasingly clear that exposure of bacterial populations to low concentrations of antibiotics has important ecological and evolutionary consequences. Low concentrations of antibiotics do not only contribute in selecting pre-existant resistant strains, but also have a non-negligible effect on the induction of phenotypic tolerance to antibiotics as well as on the emergence of new antibiotic resistances (Figure 1). Subinhibitory concentrations of antibiotics increase mutation rates, horizontal gene transfer, biofilm formation and the expression of virulence factors. Hence, the signals and the molecular mechanisms, involved in the responses to low concentrations of antibiotics, merit to be extensively studied. Targeting the key molecular actors involved in these responses or inhibiting the mutagenic pathways could indeed be a valuable strategy to increase the efficacy of antibiotic treatments and to extend the lifetime of antibiotics used in therapy, slowing down the emergence of antibiotic resistance. 
